# A Mediterranean Dietary Intervention in Female Carriers of BRCA Mutations: Results from an Italian Prospective Randomized Controlled Trial

**DOI:** 10.3390/cancers12123732

**Published:** 2020-12-11

**Authors:** Eleonora Bruno, Andreina Oliverio, Angelo Virgilio Paradiso, Antonella Daniele, Stefania Tommasi, Antonio Tufaro, Daniela Andreina Terribile, Stefano Magno, Alessio Filippone, Elisabetta Venturelli, Daniele Morelli, Ivan Baldassari, Maria Luisa Cravana, Siranoush Manoukian, Patrizia Pasanisi

**Affiliations:** 1 Department of Research, Fondazione IRCCS Istituto Nazionale dei Tumori di Milano, 20133 Milan, Italy; eleonora.bruno@istitutotumori.mi.it (E.B.); andreina.oliverio@istitutotumori.mi.it (A.O.); elisabetta.venturelli@istitutotumori.mi.it (E.V.); ivan.baldassari@istitutotumori.mi.it (I.B.); marie.louise@live.it (M.L.C.); 2 Experimental Oncology, Center for Study of Heredo-Familial Tumors, IRCCS Istituto Tumori “Giovanni Paolo II”, 70124 Bari, Italy; a.paradiso@oncologico.bari.it; 3 Experimental Oncology and Biobank Management Unit, IRCCS Istituto Tumori “Giovanni Paolo II”, 70124 Bari, Italy; antonella.daniele@oncologico.bari.it (A.D.); a.tufaro@oncologico.bari.it (A.T.); 4 Molecular Diagnostics and Pharmacogenetics Unit, IRCCS Istituto Tumori “Giovanni Paolo II”, 70124 Bari, Italy; s.tommasi@opncologico.bari.it; 5 Department of Women Health Area, Università Cattolica S. Cuore, 00168 Rome, Italy; danielaandreina.terribile@policlinicogemelli.it (D.A.T.); terapieintegrate@policlinicogemelli.it (A.F.); 6 Department of Women and Child Health and Public Health, Fondazione Policlinico Universitario A. Gemelli IRCCS, 00168 Rome, Italy; s.magno@policlinicogemelli.it; 7 Department of Diagnostic Pathology and Laboratory, Fondazione IRCCS Istituto Nazionale dei Tumori di Milano, 20133 Milan, Italy; daniele.morelli@istitutotumori.mi.it; 8 Department of Medical Oncology and Hematology, Fondazione IRCCS Istituto Nazionale dei Tumori di Milano, 20133 Milan, Italy; siranoush.manoukian@istitutotumori.mi.it

**Keywords:** randomized controlled trial, *BRCA1*/*2* mutations, mediterranean diet, insulin-like growth factor I, penetrance, personalized medicine

## Abstract

**Simple Summary:**

Unmet clinical needs in women with deleterious mutations in the BRCA1/2 genes include lifestyle recommendations as adjuvant support and risk-reducing options. Insulin-like growth factor I (IGF-I), body weight and metabolic markers of insulin resistance may affect BRCA penetrance. Therefore, we conducted a multicenter prospective two-armed randomized controlled trial to investigate whether a Mediterranean dietary intervention with moderate protein restriction reduced IGF-I and other metabolic modulators of BRCA penetrance. Out of 502 randomized women with deleterious BRCA1/2 mutations, 416 (216 in the intervention group and 200 in the control group) concluded the six-month dietary intervention and participated in the final examinations. Women in the intervention group significantly lowered serum levels of IGF-I, weight, waist circumference, hip circumference, total cholesterol and triglycerides with respect to the control group. Our findings suggest that a Mediterranean diet with moderate protein restriction is effective in reducing potential modulators of BRCA penetrance.

**Abstract:**

*Background:* Women carriers of *BRCA1*/*2* mutations face a high lifetime risk (penetrance) of developing breast and/or ovarian cancer. Insulin-like growth factor I (IGF-I), body weight and markers of insulin resistance affect BRCA penetrance. We conducted a multicenter prospective two-armed (1:1) randomized controlled trial (NCT03066856) to investigate whether a Mediterranean dietary intervention with moderate protein restriction reduces IGF-I and other metabolic modulators of BRCA penetrance. *Methods*: BRCA carriers, with or without a previous cancer, aged 18–70 years and without metastases were randomly assigned to an active dietary intervention group (IG) or to a control group (CG). The primary endpoint of the intervention was the IGF-I reduction. *Results*: 416 women (216 in the IG and 200 in the CG) concluded the six-month dietary intervention. The IG showed significantly lowered serum levels of IGF-I (−11.3 ng/mL versus −1.3 ng/mL, *p* = 0.02), weight (−1.5 Kg versus −0.5 Kg, *p* < 0.001), waist circumference (−2 cm versus −0.7 cm, *p* = 0.01), hip circumference (−1.6 cm versus −0.5 cm, *p* = 0.01), total cholesterol (−10.2 mg/dL versus −3.6 mg/dL, *p* = 0.04) and triglycerides (−8.7 mg/dL versus + 5.5 mg/dL, *p* = 0.01) with respect to the CG. *Conclusions*: A Mediterranean dietary intervention with moderate protein restriction is effective in reducing IGF-I and other potential modulators of BRCA penetrance.

## 1. Introduction

Women with a deleterious mutation in the *BRCA1*/*2* genes face a lifetime risk (penetrance) of developing breast cancer (BC) in the order of 55%, compared to 12% in the general population, and of developing ovarian cancer (OC) in the order of 16–59% [1,2,3]. A more recent prospective study of 10,000 female carriers of *BRCA1*/*2* mutations [4] showed a 72% chance of developing BC by age 80 (95% CI, 65–79%) for *BRCA1* and a 69% chance (95% CI, 61–77%) for *BRCA2* mutation carriers, respectively. The lifetime risk of developing OC was 44% (95% CI, 36–55%) for *BRCA1* and 17% (95% CI, 11–25%) for *BRCA2* mutation carriers [4]. Given the incomplete penetrance, our hypothesis is that other factors, genetic (polymorphisms) and/or “environmental”, influence the cancer risk.

Cancer risk is higher if genotype carriers are obese or have high energy intake and life-long weight gain [5,6]. Obesity may affect BRCA penetrance through a number of mechanisms, including insulin resistance and insulin-like growth factor I (IGF-I) regulation. Women with *BRCA1*/*2* mutations more frequently develop type-2 diabetes after a BC diagnosis [7] compared with carriers without cancer. The prediabetic condition, when the levels of insulin and growth factors are typically very high, can raise the risk of BC in these women. Consistently, retrospective case-control analyses suggested that IGF-I and metabolic syndrome (MS) were associated with an increased BRCA penetrance [8,9].

We conducted a randomized controlled trial [10,11,12,13,14] of women with deleterious *BRCA1*/*2* mutations to test whether a Mediterranean dietary intervention with moderate protein restriction significantly reduces IGF-I and other potential modulators of penetrance. 

This paper reports the final results of the trial.

## 2. Results 

**Figure 1:**
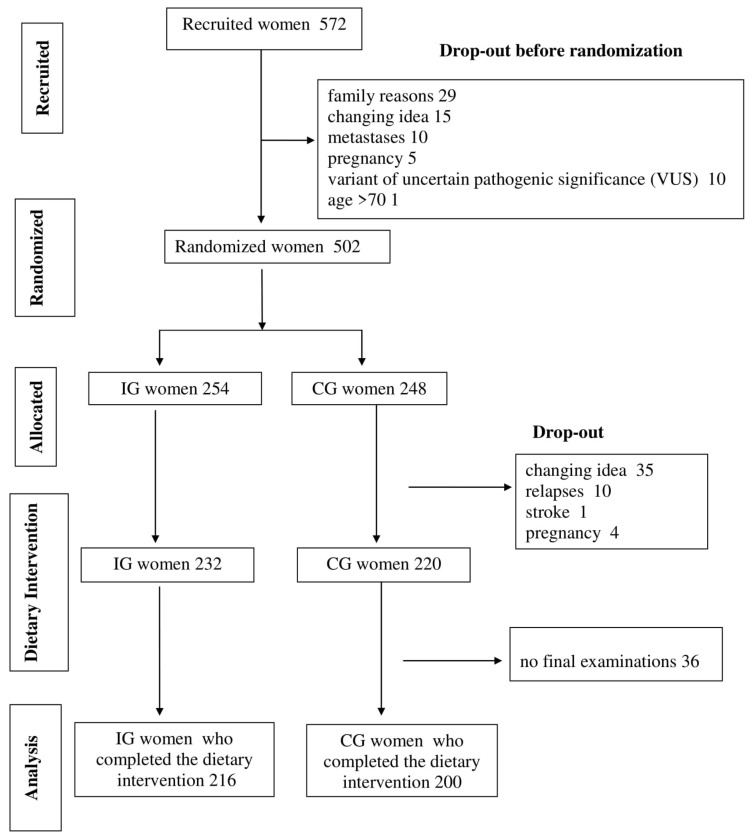
A flow chart of the trial. The flow chart, following Consolidated Standards of Reporting Trials CONSORT guidelines, describes the progress through the phases of our two-arm randomized controlled trial (recruitment, randomization, intervention allocation, dietary intervention and data analysis).

A total of 572 women attended the recruitment meetings and signed informed consent forms (Figure 1).

Seventy chose not to proceed before the randomization and “family reasons” was the leading reason for dropping out (42%). Therefore, 502 women with *BRCA1*/*2* mutations (198 unaffected and 304 affected) were randomized, 254 in the intervention group (IG) and 248 in the control group (CG). 

Table 1 reports the general characteristics of the trial population by randomization group.

IG women were slightly older, with fewer current smokers (*p* = 0.27). They had a nonsignificantly higher frequency of natural menopause (*p* = 0.37) and of previous preventive salpingo-oophorectomy (*p* = 0.51). The distribution of previous cancer, type of cancer, age at diagnosis and years from cancer diagnosis were similar. Among BCs, the proportions of estrogen receptor (ER)-negative tumors and of the axillary node metastases were homogeneous. However, the distribution of *BRCA1*/*2* mutations significantly differed in the two groups (*p* = 0.01) (Table 1). Current cancer hormonal treatment was homogeneous between the two randomized groups (*p* = 0.65).

At baseline, IG women presented a nonsignificantly slightly lower BMI and waist circumference (respectively *p* = 0.07 and *p* = 0.10) and a significantly smaller hip circumference (*p* = 0.01). The two randomized groups were homogeneous as regards metabolic parameters and serum levels of insulin and IGF-I (data not shown).

As for their 24-h food frequency diaries on baseline food intake (Table S1), the CG had slightly higher, although nonsignificantly, consumption of sugary food and beverages (about 2 times/day vs. 1.6 times/day in the IG, *p* = 0.05), legumes/soy products (about 0.4 times/day vs. 0.3 times/day in the IG, *p* = 0.11) and red/processed meat (about 0.5 time/day vs. 0.4 times/day in the IG, *p* = 0.06). Overall, as regards 24-h food frequency diaries, the two groups were substantially homogeneous. Consistently, intervention and control women reported similar Mediterranean food consumption, with total Mediterranean Diet Adherence Screener (MEDAS) scores of (on average) 7.2 and 7.1 (*p* = 0.54), respectively. 

Out of 502 participants, 416 women (83%), 216 in the IG and 200 in the CG, concluded the six-month dietary intervention and were available for the final examinations. After randomization, 4 women became pregnant (2 in the IG and 2 in the CG), 10 relapsed (6 in the IG and 4 in the CG), 1 had a stroke (in the IG) and 35 changed their mind for family/work reasons (13 in the IG and 22 in the CG), thus effectively dropping from the study without any dietary intervention. Another 36 women (16 in the IG and 20 in the CG) partially complied with the nutritional activities but did not turn up for the final examinations. Overall, the proportions of dropout were 15% in the IG and 19% in the CG.

In both groups, most of the anthropometric and metabolic parameters significantly improved at the end of the six-month dietary intervention (Table 2). However, control women did not show any significant reduction in hip circumference or total cholesterol and their triglycerides rose. The CG had no substantial changes in IGF-I (−1.3 ng/mL, *p* = 0.67) while the IG showed a significant reduction (−11.3 ng/mL, *p* < 0.001). Both groups showed a significant reduction in insulin levels (−5.3 μIU/mL, *p* < 0.001 in the CG and −7.7 μIU/mL, *p* < 0.001 in the IG). 

The results of the “delta” analysis of differences between the two groups (intention-to-treat analysis), controlling for center, age, education, BMI at baseline and menstrual status, showed that the IG lost significantly more weight (*p* < 0.001) and BMI (*p* < 0.001) and lowered waist circumference (*p* = 0.01), hip circumference (*p* = 0.01), total cholesterol (*p* = 0.04) and triglycerides (*p* = 0.01) significantly more than the CG (Table 2). The IG significantly lowered IGF-I levels (*p* = 0.02) with respect to the CG (Table 2). Repeating the analyses on metabolic factors and IGF-I after further controlling for weight change, the results continued to show significant reductions in triglycerides (*p* = 0.02) and IGF-I (*p* = 0.03), while the reduction in total cholesterol became borderline significant (*p* = 0.05).

The results did not change after controlling for the baseline value of the variable under study. They did not differ after stratifying for type of BRCA mutation (*BRCA1* or *BRCA2*).

**Figure 2:**
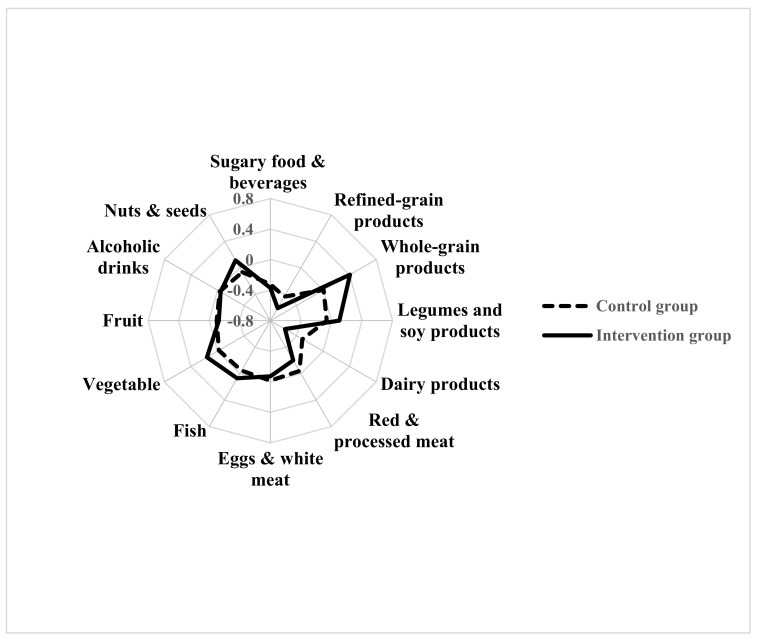
Changes in frequencies of food consumption by randomization group (intention-to-treat analysis, 416 women).

Compared to the CG, the IG significantly improved their consumption of the recommended food, i.e., whole grain products (*p* < 0.001), legumes (*p* = 0.02) and nuts and seeds (*p* = 0.02), and significantly reduced their consumption of dairy products (*p* = 0.01) and of red and processed meat (*p* = 0.04) (Figure 2). 

The distribution of the “Delta, Δ” changes in frequencies of food consumption in the IG and in the CG is represented by a Kiviat diagram. This graphic representation consists of a sequence of rays which originate from a center and form equal angles to each other; each ray represents one food/food group. The distance from the center of the point marked on the radius is the “Delta, Δ” change of frequencies of food/food group consumption. The points on the rays are joined with two segments, continuous for the IG and dashed for the CG.

Adjusted multiple regression analysis suggested that the reduction in animal products consumption was directly related, although nonsignificantly, to the reduction of IGF-I levels (*p* = 0.09) and randomization group (*p* = 0.01). Furthermore, in the multinomial logistic regression analysis on the whole population, women who experienced the higher reduction in animal products (at least –1 frequency/day of animal products consumption) showed an Odds Ratio OR of 1.21 (1.04–1.41 95% CI), placing them in the higher tertile of reduction of IGF-I (Table 3). These results were significant only in the IG with an OR of 1.32 (1.08–1.62 95% CI) vs. an OR of 1.10 (0.86–1.42 95% CI) in the CG.

We did not observe any significant associations involving the reduction of legumes, refined products consumption or body weight.

The “delta” analysis of differences in the consumption of Mediterranean food (MEDAS data) confirmed previous food’s results. Compared to the CG, intervention women had a significantly larger improvement in the MEDAS score (Table S2).

## 3. Discussion

Findings from this Italian randomized controlled trial show that a dietary intervention based on the MedDiet with moderate protein restriction is effective in reducing IGF-I and other potential modulators of BRCA penetrance. Women in the IG significantly increased their adherence to the MedDiet, enjoyed substantial improvements in their metabolic and anthropometric parameters and showed a significant decrease in the serum levels of IGF-I with respect to the CG. 

The reduction of IGF-I was the primary endpoint of our trial. Previous studies found that IGF-I and lifestyle factors linked to the insulin-IGF-I axis, such as body weight, high energy intake, milk intake and low levels of physical activity, were associated with a higher BRCA penetrance [5,6,9,15,16]. However, these associations mainly come from retrospective analyses and the results might be strongly distorted by bias (i.e., temporal, recall, survival). The follow-up of this trial cohort, with prospective evaluation of the data we collected, will give us the opportunity of studying the “environmental” modulators of BRCA penetrance and their impact in the natural history of BRCA-related cancer. 

Our results also suggest that control women reported changes in some parameters of interest. These improvements were partially expected, by design. In fact, all the women, on the day they were recruited, were given general lifestyle recommendations (the World Cancer Research Fund/American Institute for Cancer Research WCRF/AICR decalogue) and received a standard meal that respected WCRF principles [17]. These recommendations and the attention of women carriers of *BRCA* mutations to the benefits of a healthy lifestyle probably led the CG to make some adjustments in their dietary habits. The CG, however, presented no changes in body weight, cholesterol, triglycerides or IGF-I: calorie and especially protein restriction are required to reduce IGF-I and only participation in the specific nutritional activities fostered the change.

Previous trials tried to act on IGF-I through an insulin-lowering diet, succeeding in reducing IGF-I bioavailability by increasing the liver synthesis of IGF-binding proteins 1 and 2, but this did not lower the serum levels [18,19,20]. Reducing protein intake to 0.9 g per kg per day markedly lowered the IGF-I concentration, suggesting that calorie restriction does not reduce IGF-I unless protein intake is reduced as well [21]. In the present trial, our broad changes in diet aimed at reducing calorie intake through the reduction of high glycaemic/insulinemic index food and saturated fat sources and reducing protein intake through a significant reduction of animal products consumption, thus lowering serum levels of IGF-I. The entries from the 24-h food frequency diaries indicate that intervention women markedly reduced their intake of dairy and total animal products and high calorie-dense food (sugars and refined products) and ate more satiating food (whole grain products and legumes). Data from the MEDAS questionnaires confirm these changes in the dietary habits of intervention women and show their increased adherence to the MedDiet. The IG experienced greater dietary changes compared to the CG and achieved greater reductions in body weight, metabolic parameters and IGF-I levels. Our dietary instruments did not allow measurement of the women’s nutrients intake and the frequencies/day of consumption from our 24-h diaries are only proxies of the absolute intake. Therefore, our findings might be related to a reduced energy intake, a reduced protein intake, an increase in MEDAS score or a combination of these factors. However, when we studied the association between the reduction of serum levels of IGF-I and the reductions in body weight and in several food consumptions, our results suggested a direct relationship between the reduction in animal products and the reduction in IGF-I. In fact, compared to women whose consumption of animal products did not change or got worse, women who reduced at least 1 frequency/day of animal products enjoyed a larger reduction of IGF-I. 

In the intention-to-treat analysis, the reduction of IGF-I levels in the intervention women compared to the CG persisted at a significant level after further adjustment for weight loss. This finding and the above described results about the association with food consumptions suggest that the change in IGF-I was due more to the composition of the diet than just the lower calorie intake. 

Women with deleterious mutations in *BRCA1/2* often spend a large part of their life dealing with their genetic risk of cancer. Despite ad hoc surveys and the strong protection offered by prophylactic surgery, acceptance of these options varies among individuals and by country. Women often inquire about less invasive risk-reducing options, including dietary and physical activity recommendations, and physicians need to be able to give them evidence-based answers regarding their lifestyle choices, especially for those who opt not to undergo surgery or chemoprevention. The present results and our previous findings [10,11,13] indicate that women carriers, if properly instructed, are capable of substantially improving their dietary habits, with significant reduction in their potential modulators of BRCA penetrance. In Germany, the LIBRE study has concluded its feasibility phase (LIBRE-1) confirming the practicability of an intervention involving Mediterranean diet and physical activity in BRCA mutation carriers [22,23]. Women from the German cohort increased their MEDAS score after a 12-month intervention with similar improvements of our cohort. The prospective evaluation of the German cohort (LIBRE-2) is ongoing. The future results of the Italian and the German studies will allow us to clarify the role of lifestyle in BRCA penetrance.

Our trial has some limitations. At baseline, there were some differences in BMI, hip circumference and the distribution of *BRCA1/2* mutations between the IG and CG. This was probably due to the fact that the randomization, for ethical and practical reasons, was balanced for family relationship and women belonging to the same family were randomized in the same randomization group by study protocol. However, repeating the analyses controlling for the baseline value of the variable under study did not change the overall results. Furthermore, results did not differ when stratifying the analysis for type of mutation (*BRCA1* or *BRCA2*). Actually, we did not expect a different response to the dietary intervention on the basis of the type of mutation. 

The baseline differences between the IG and CG did not involve IGF-I and metabolic parameters. 

One limitation of this study was that without a prospective evaluation we could not quantify how the reduction of serum IGF-I affected the BRCA-related cancer risk. Prospective studies have investigated the association between IGF-I and the risk of sporadic BC [24]. By considering the relative risk of BC reported by our research group in the ORDET Italian cohort [25], we can estimate that the ~11% reduction of serum levels of IGF-I that we obtained through the dietary intervention corresponds to a reduction in the BC risk in the order of 10–20%. This is only an estimate about the potential protective effect of the reduction of IGF-I because no prospective studies exist in this context on BRCA mutation carriers. By following up our trial cohort over time we hope to demonstrate a reduction in BRCA-related cancer risk, or less progressive disease, and lower cancer mortality rates in women adhering to a healthy lifestyle. 

Another limitation was that intervention women had lower serum insulin but the change compared to CG was not significant. There was a similar effect for plasma glucose. The general improvements of the CG and their reductions in consumption of refined foods and sweets might well explain this. Furthermore, to help women reduce their animal protein intake, we encouraged the consumption of whole-grain cereals, legumes and vegetables, thus probably increasing the share of complex carbohydrates in their diet. Carbohydrates contributed 55–60% of total calories in our menus.

A further limitation was that, although the IG were given notes on the nutritional content of the menus proposed and recipes to be cooked at home, we did not use any dietary instruments to estimate the women’s nutrient intake. However, the 24-h diaries were extremely efficient for describing and comparing the average consumption of specific foods and dietary patterns between populations, in line with our aim [26].

Increasing attention is being paid to BRCA-related cancer. However, lifestyle intervention studies in women carriers of mutations in *BRCA1/2* are still rare and this Italian study is currently the largest prospective two-arm dietary randomized controlled trial with results. Further analyses and the follow-up of the cohort will help in developing practical and safe prevention interventions to lower the number of cases and deaths attributable to *BRCA1*/*2* mutations.

## 4. Patients and Methods

### 4.1. Study and Subjects

This Italian multicenter prospective two-armed randomized (1:1) controlled trial (reference: NCT03066856) aimed at investigating whether an active dietary intervention based on the Mediterranean diet (MedDiet) with moderate protein restriction—mainly animal protein, down to ~11% of total calorie intake—significantly reduced IGF-I and metabolic modulators of BRCA penetrance. Detailed information regarding the study design and preliminary results have been previously reported [9,10,11,12]. Briefly, eligible study subjects were women aged 18–70, with or without a previous diagnosis of BC/OC, without metastases, who were found to be carriers of deleterious *BRCA1*/*2* mutations. Unaffected women with bilateral prophylactic mastectomy were not included in the trial cohort. All participants were fully informed about the study, signed a written informed consent form and received general recommendations for the prevention of cancer (the WCRF/AICR decalogue) [17]. After the baseline examinations, women were randomly assigned to an active dietary intervention group (IG) or to a control group (CG) that carried on following the baseline recommendations. Center, age (≤40 and >40 years) and family relationship were considered to balance the randomization. 

The trial was approved by the Ethics Committee of the coordinating center, the Fondazione IRCCS Istituto Nazionale dei Tumori di Milano (approval number: INT106/13). 

### 4.2. Data Collection and Measurements

Data collection and measurements were performed in each study center and recorded in a central electronic database provided at the Fondazione IRCCS Istituto Nazionale dei Tumori di Milano. 

Women were asked:

- to complete a questionnaire on their medical history and major cancer risk factors (reproductive and behavioral factors);
- to attend a clinic for anthropometric measurements. Height and body weight were collected without shoes and heavy clothes. Waist circumference was measured with a professional measuring tape at natural waist or, if not identifiable, at the midpoint between the iliac crest and the lower rib according to standard techniques. Hip circumference was measured at the level of the greater throcanter. Blood pressure was measured using an electronic sphygmomanometer;
- to give a 20-mL blood sample for metabolic/hormonal assays;
- to provide information on their health and to allow the study officials to contact their usual physicians, to consult clinical notes and to examine biopsy material, as necessary;
- to fill in a 24-h food frequency diary of each previous day’s food intake (65 food items) and the validated 14-point Mediterranean Diet Adherence Screener [27]. The 24-h food frequency diary contained a list of 65 food items. The diary did not include information on portion size or weight, nor on recipes. Women had to indicate only whether, the previous day, they had or not eaten the specified food at breakfast, lunch, dinner and breaks. MEDAS [27] consists of 14 questions on food consumption frequency and two on eating habits.

Anthropometric and body composition measurements, blood samples and dietary data were provided at baseline and at the end of the six-month dietary intervention. 

### 4.3. Dietary Intervention

Women randomized in the IG were invited to attend six full days of dietary activities, involving six cookery courses followed by lunch and six nutritional conferences, over the following six months. The proposed diet (Figure S1), mainly based on MedDiet principles and recipes, and including some fermented food from the Japanese tradition (miso, soy sauce, tempeh, humeboshi) to facilitate digestion and serve as a reliable reservoir for microbiota [28], aimed to:

- reduce the overall consumption of animal food in order to lower protein intake to ~11% of total calorie intake. Animal milk, associated with higher plasma levels of IGF-I [29], was markedly limited. The use of plant-based unsweetened milk and creams was encouraged. Among animal food, fish, especially cold-water fish (e.g., salmon and mackerel), rich in omega-3 poly-unsaturated fatty acids, was privileged. The lower safe limit of protein intake for lean adults is about 0.6 g of protein per kg body weight; to ensure a 25% margin of safety, the current recommended lower limit of protein intake has been set at 0.75 g/kg body weight [30,31]. Our goal was ~0.9–1.1 g/kg body weight. IG women received the menus supplied during cookery classes, recipes and handouts with illustrations of the food portions, especially of food with high protein content;
- markedly reduce the consumption of refined foods, such as sugar, sugary drinks, refined food, potatoes and sweets, that promote pro-inflammatory cytokines. The consumption of whole-grain food was encouraged in order to attenuate the postprandial glucose response and to acutely improve insulin homeostasis [32]. Desserts were prepared without adding sugars, using dried fruits. Nuts and legume flours were proposed to prepare sweets and for savory cookery recipes;
- help participants distinguish between the different types of fat in foods (saturated, trans-, mono- and poly-unsaturated). Processed and red meat (rich in saturated fats), but also some food of vegetable origin, such as margarine (which contains trans-fatty acids), promote inflammation and needed to be substantially reduced. Cold-pressed extra-virgin olive oil was the main source of fat;
- encourage an improvement of dietary habits, as defined by macro- and micronutrient needs.
- Nutritional requirements, menus and recipes were standardized among study centers.

### 4.4. Laboratory Methods

Blood samples were collected 90 min after a standard meal (vegetable soup; 50 g, before cooking, of brown rice seasoned with seeds and salt; vegetables; and 30 g, before cooking, of legumes) to identify early signs of insulin resistance. The samples were aliquoted and stored at −80 °C. Plasma glucose, triglycerides and total LDL and HDL cholesterol were measured using routine laboratory techniques. IGF-I and insulin analyses were done centrally at the Fondazione IRCCS Istituto Nazionale dei Tumori di Milano. Serum IGF-I was measured using commercial radioimmunoassay kits from Biosource (Nivelles, Belgium), obtaining intra- and inter-assay coefficients of variation of 2.6% and 10.8%, respectively, for a mean IGF-I value of 193.7 ng/mL. The insulin dosage was performed using an immunoradiometric kit from Immunotech (Prague, Czech Republic). The kit showed intra- and inter-assay coefficients of variation of 2.2% and 5.1%, respectively, for a mean insulin value of 10 μIU/mL.

The technicians analyzing the serum samples were blinded to the patients’ randomization arm.

### 4.5. Statistical Analysis 

The women’s general characteristics were summarized by randomization group using frequencies or means and standard deviation (SD) and compared using χ^2^ or *t*-tests, as appropriate. 

The Wilcoxon rank sum test was used to compare frequencies of food consumptions. We collected the entries from the 24-h diaries of the previous day’s intake of single food items and food groups. Food group variables were generated by summing single food items: sugary food and beverages (white sugar + brown sugar + malt + chocolate + candies + sugary beverages); refined grain products (white bread + white rice + egg noodles + biscuits + corn flakes + sweetened muesli); whole-grain products (whole bread + whole rice + other whole grain cereals + unsweetened muesli + oat flakes); legumes and soy products (legumes + tofu/tempeh); dairy products (milk + other dairy products); red/ processed meat (red + processed meat); other animal products (white meat + eggs); alcoholic beverages (wine + beer + spirits). 

As for the short MEDAS, the total score depended on the answer to each item (14 items), each rated 0 or 1. If the condition was not met, 0 points were recorded for the category. The final MEDAS score, ranging from 0 to 14, was the sum of all the points. A two-sample *t*-test was used to compare baseline and six-month measurements. 

The primary endpoint of the study was the IGF-I reduction. The original statistical plan provided for the randomization of 600 women with *BRCA1*/*2* mutations assuming that the dietary intervention might lower IGF-I by 10% on average with a 96% statistical power. After the ad interim analysis, an amendment to the original protocol was provided and accepted by AIRC (IG 2015–17,151), reducing the sample size to 500 randomized women. With more than 464 participants, the overall study provided 85% power in two-sided tests with a type-I error rate of 5% to detect a between-group difference in IGF-I reduction of 11%. 

We used an analysis of variance (ANOVA)-based approach to examine the effect of the dietary intervention on IGF-I and the other variables of interest at their original continuous scale. We checked first for interactions between the two independent variables (randomization group and time) and the dependent variable (factors under study). We therefore analyzed the magnitude of changes in the variables (response variable) using the difference (delta, Δ) between the end of the study and baseline values for each woman in the two groups, and controlled for center, age (quintiles), education (first, second and third level), BMI at baseline (quintiles) and menstrual status (still menstruated, natural menopause, induced menopause). Since the appropriate way to estimate the difference in change from baseline is to include the baseline measurement of the variable as covariate [33], we repeated the analyses taking into account this further adjustment. 

The association between the reduction in IGF-I (Δ IGF-I in tertiles) and the reduction of consumption of animal products, legumes, refined products and Δ body weight was studied with a multinomial logistic regression model and 95% confidence intervals (CI) within the total population at the end of the dietary intervention. Center, age, education, BMI at baseline, menstrual status and randomization group were used as model covariates. 

A *p*-value of < 0.05 was taken as significant. All statistical tests were two-sided. Analyses were done using the STATA 14 statistical package (StataCorp, College Station, TX, USA).

## 5. Conclusions

Unmet clinical needs in women with deleterious mutations in the *BRCA1*/*2* genes include lifestyle recommendations as adjuvant support and risk-reducing options. 

Our findings suggest that women carriers, if properly instructed, are capable of increasing their adherence to the MedDiet, of enjoying substantial improvements in their metabolic and anthropometric parameters and of significantly lowering serum levels of IGF-I. 

This Italian study is currently the largest prospective two-arm dietary randomized controlled trial in women carriers of *BRCA1/2* mutations and these results are promising. Further analyses and the follow-up of the cohort will help in developing practical and safe prevention interventions in *BRCA* families.

## Figures and Tables

**Table 1:** Baseline characteristics of the study population (502 women).

Mean ± SD	IG (254)	CG (248)
Age (yrs)	47.6 ± 10.8	46.0 ± 11.1
Menarche (yrs)	12.4 ± 1.4	12.4 ± 1.6
Age at first live birth (yrs)	29.0 ± 5.9	28.9 ± 4.7
Age at first diagnosis (yrs) (if affected)	44.1 ± 8.4	43.2 ± 8.8
Time from cancer diagnosis (yrs) (if affected)	6.4 ± 7.4	6.4± 6.8
Education (%)		
First level	16.9	16.9
Second level	44.1	43.6
Third level	39	39.5
Pregnancy (%)		
Yes	71.3	70.8
Number of children		
≤2	85.1	84.9
≥3	14.9	15.1
Menopause (%)	73.9	72.6
Natural menopause (%)	26.2	22.2
Preventive salpingo-oophorectomy (%)	25.6	22
Oral contraceptive in the past (%)	66.5	67.2
Current smoking (%)	12.1	13
Physical Activity (%)		
None	36.3	36.2
Moderate or Vigorous	48	46.8
Moderate and Vigorous	15.7	17
Mutated gene (%)		
*BRCA1*	56.7	68.9
*BRCA2*	43.3	31.1
Cancer type if affected (%)		
Breast	81.4	81.1
Breast and ovary	5.8	2
Ovary	12.8	16.9
Infiltrating duct BC (%)	82.2	87.4
ER-negative (%)	44.6	41.2
Axillary node metastasis (%)	25.6	26.2
Cancer hormonal treatment (%)	46.8	50.4
Current cancer hormonal treatment (%)	26.9	25.7

IG, intervention group; CG, control group; BC, breast cancer; ER, estrogen receptor.

**Table 2:** Results of before–after analysis and intention-to-treat analysis (416 women).

Variable	IG (216)		CG (200)			IG (216)		CG (200)	*p* **
	Baseline Mean ± SD	Six Months Mean ± SD	*p* *	Baseline Mean ± SD	Six Months Mean ± SD	*p* *	Δ of Differences	Δ of Differences	
Weight (Kg)	62.1 ± 10.7	60.6 ± 10.8	<0.001	65.6 ± 14.6	65.1 ± 14.5	0.01	−1.5	−0.5	<0.001
BMI (kg/m^2^)	23.9 ± 4.4	23.3 ± 4.3	<0.001	24.7 ± 5.1	24.5 ± 5.0	0.01	−0.6	−0.2	<0.001
Waist circ. (cm)	77.1 ± 11.7	75.1 ± 10.2	<0.001	79.0 ± 13.5	78.3 ± 13.0	0.02	−2.0	−0.7	0.01
Hip circ. (cm)	98.6 ± 9.2	97.0 ± 9.1	<0.001	101.0 ± 10.4	100.4 ± 10.5	0.09	−1.6	−0.5	0.01
Systolic press. (mmHg)	125.9 ± 17.8	122.7 ± 14.3	0.002	124.9 ± 15.1	121.3 ± 14.1	0.003	−3.2	−3.6	0.53
Diastolic press. (mmHg)	82.0 ± 11.0	79.9 ±10.9	0.005	81.4 ± 10.3	79.5 ± 9.2	0.01	−2.2	−1.9	0.74
Glycemia (mg/dL)	101.2 ± 22.0	93.8 ± 18.3	<0.001	101.4 ± 24.5	92.5 ± 19.5	<0.001	−7.4	−8.8	0.51
Total cholesterol (mg/dL)	199.2 ± 38.3	189.0 ± 33.5	<0.001	198.9 ± 37.8	195.3 ± 38.5	0.09	−10.2	−3.6	0.04
HDL^+^ cholesterol (mg/dL)	68.5 ± 16.0	66.6 ± 15.2	0.03	69.4 ± 18.0	69.7 ± 20.1	0.79	−1.9	+0.3	0.21
LDL^++^ cholesterol (mg/dL)	117.0 ± 35.6	111.4 ± 32.9	<0.001	117.2 ± 35.0	112.1 ± 34.0	0.01	−5.6	−5.1	0.82
Triglycerides (mg/dL)	105.1 ± 71.0	96.4 ± 48.0	0.04	101.6 ± 57.7	107.1 ± 60.6	0.07	−8.7	+5.5	0.01
IGF-I (ng/mL)	178.9 ± 67.7	167.6 ± 72.0	<0.001	173.1 ± 64.6	171.8 ± 62.4	0.67	−11.3	−1.3	0.02
Insulin (µIU/mL)	21.3 ± 18.7	13.5 ± 11.6	<0.001	20.2± 16.9	14.7 ± 12.2	<0.001	−7.7	−5.5	0.11

* *p* of *t*-test for difference between baseline and six months by group. ** *p* of ANOVA controlling for center, age, education, BMI at baseline and menstrual status. HDL^+^ = High Density Lipoprotein, LDL^++^ = Low Density Lipoprotein.

**Table 3:** Adjusted ORs (95% CIs) of reduction of IGF-I (ΔIGF-I, tertiles) according to the reduction of body weight (Δ Weight), animal products, legumes and refined products consumption.

Δ IGF-I	Δ Weight	Δ Animal Products	Δ Legumes	Δ Refined Products
1st tertile (≥9.7 ng/mL)	1	1	1	1
2nd tertile (9.6 to −22.2 ng/mL)	1.01 (0.93–1.10)	1.18 * (1.0–1.36)	0.89 (0.64–1.24)	1.12 (0.95–1.34)
3rd tertile (> −22.3 ng/mL)	1.04 (0.95–1.14)	1.21 * (1.04–1.41)	0.97 (0.70–1.35)	1.05 (0.88–1.26)

* significant results.

## Supplementary Materials

The following are available online at https://www.mdpi.com/2072-6694/12/12/3732/s1, Figure S1: An example of the proposed menu, Table S1: Baseline daily frequency of consumption of selected food or food groups by randomization group, Table S2: Analysis of differences (intention-to-treat analysis) in food consumption between the two groups (416 women).

## Project Administration

P. Pasanisi, A. Paradiso and D.A Terribile; Funding Acquisition: P. Pasanisi.

## References

[1] Antoniou A., Pharoah P.D., Narod S., Risch H.A., Eyfjord J.E., Hopper J.L., Loman N., Olsson H., Johannsson O., Borg Å. (2003). Average risks of breast and ovarian cancer associated with BRCA1 or BRCA2 mutations detected in case Series unselected for family history: A combined analysis of 22 studies. Am. J. Hum. Genet..

[2] Berrino J., Berrino F., Francisci S., Peissel B., Azzollini J., Pensotti V., Radice P., Pasanisi P., Manoukian S. (2015). Estimate of the penetrance of BRCA mutation and the COS software for the assessment of BRCA mutation probability. Fam.Cancer.

[3] Lalloo F., Evans D.G. (2012). Familial breast cancer. Clin. Genet..

[4] Kuchenbaecker K.B., Hopper J.L., Barnes D.R., Phillips K.A., Mooij T.M., Roos-Blom M.J., Jervis S., Van Leeuwen F.E., Milne R.L., Andrieu N. (2017). Risks of breast, ovarian, and contralateral breast cancer for brca1 and brca2 mutation carriers. JAMA.

[5] Nkondjock A., Ghadirian P. (2004). Epidemiology of breast cancer among BRCA mutation carriers: An overview. Cancer Lett..

[6] Pettapiece-Phillips R., Narod S.A., Kotsopoulos J. (2015). The role of body size and physical activity on the risk of breast cancer in BRCA mutation carriers. Cancer Causes Control..

[7] Bordeleau L., Lipscombe L., Lubinski J., Ghadirian P., Foulkes W.D., Neuhausen S., Ainsworth P., Pollak M., Sun P., Narod S.A. (2011). Diabetes and breast cancer among women with BRCA1 and BRCA2 mutations. Cancer.

[8] Dumais V., Lumingu J., Bedard M., Paquet L., Verma S., Fontaine-Bisson B. (2017). Prevalence of insulin resistance, metabolic syndrome, and type 2 diabetes in Canadian women at high risk for breast cancer. Breast J..

[9] Pasanisi P., Bruno E., Venturelli E., Manoukian S., Barile M., Peissel B., De Giacomi C., Bonanni B., Berrino J., Berrino F. (2011). Serum levels of IGF-I and BRCA penetrance: A case control study in breast cancer families. Fam. Cancer..

[10] Bruno E., Manoukian S., Venturelli E., Oliverio A., Rovera F., Iula G., Morelli D., Peissel B., Azzolini J., Roveda E. (2018). Adherence to mediterranean diet and metabolic syndrome in BRCA mutation carriers. Integr. Cancer Ther..

[11] Daniele A., Paradiso A.V., Divella R., Digennaro M., Patruno M., Tommasi S., Pilato B., Tufaro A., Barone M., Minoia C. (2020). The role of circulating adiponectin and SNP276G>T at ADIPOQ Gene in BRCA-mutant women. Cancer Genom. Proteom..

[12] Pasanisi P., Bruno E., Manoukian S., Berrino F. (2014). A randomized controlled trial of diet and physical activity in BRCA mutation carriers. Fam.Cancer.

[13] Pasanisi P., Bruno E., Venturelli EMorelli D., Oliverio A., Baldassari I., Rovera F., Iula G., Taborelli M., Peissel B., Azzollini J. (2018). A dietary intervention to lower serum levels of IGF-I in BRCA mutation carriers. Cancers (Basel).

[14] Bruno E., Oliverio A., Paradiso A., Daniele A., Tommasi S., Terribile D.A., Filippone A., Digennaro M., Pilato B., Danza K. (2020). Lifestyle characteristics in BRCA-mutant women: Results from an Italian trial cohort. Clin. Breast Cancer.

[15] Bissonauth V., Shatenstein B., Fafard E., Maugard C., Robidoux A., Narod S., Ghadirian P. (2009). Weight history, smoking, physical activity and breast cancer risk among French-Canadian women non-carriers of more frequent BRCA1/2 mutations. J. Cancer Epidemiol..

[16] Pasanisi P., Berrino J., Fusconi E., Curtosi P., Berrino F. (2005). European case-only study (COS) on familial breast cancer. J. Nutr..

[17] World Cancer Research Fund, American Institute for Cancer Research (2007). Food, Nutrition, Physical activity and the Prevention of Cancer: A Global Perspective.

[18] Berrino F., Bellati C., Secreto G., Amerini E., Pala V., Panico S., Allegro G., Kaaks R. (2001). Reducing bioavailable sex hormones through a comprehensive change in diet: The diet and androgens (DIANA) randomized trial. Cancer Epidemiol. Biomarkers Prev..

[19] Fontana L., Villareal D.T., Das S.K., Smith S.R., Meydani S.N., Pittas A.G., Klein S., Bhapkar M., Rochon J., Ravussin E. (2016). Effects of 2-year calorie restriction on circulating levels of IGF-1, IGF-binding proteins and cortisol in nonobese men and women: A randomized clinical trial. Aging Cell.

[20] Kaaks R., Bellati C., Venturelli E., Rinaldi S., Secreto G., Biessy C., Pala V., Sieri S., Berrino F. (2003). Effects of dietary intervention on IGF-I and IGF-binding proteins, and related alterations in sex steroid metabolism: The diet and androgens (DIANA) randomised trial. Eur. J. Clin. Nutr..

[21] Fontana L., Weiss E.P., Villareal D.T., Klein S., Holloszy J.O. (2008). Long-term effects of calorie or protein restriction on serum IGF-1 and IGFBP-3 concentration in humans. Aging Cell..

[22] Kiechle M., Dukatz R., Yahiaoui-Doktor M., Berling A., Basrai M., Staiger V., Niederberger U., Marter N., Lammert J., Grill S. (2017). Feasibility of structured endurance training and Mediterranean diet in *BRCA1* and *BRCA2* mutation carriers—An interventional randomized controlled multicenter trial (LIBRE-1). BMC Cancer..

[23] Seethaler B., Basrai M., Vetter WLehnert K., Engel C., Siniatchkin M., Halle M., Kiechle M., Bischoff S.C. (2020). Fatty acid profiles in erythrocyte membranes following the Mediterranean diet—data from a multicenter lifestyle intervention study in women with hereditary breast cancer (LIBRE). Clin. Nutr..

[24] Key T.J., Appleby P.N., Reeves G.K., Roddam A.W., Endogenous Hormones and Breast Cancer Collaborative Group (2010). Insulin-like growth factor 1 (IGF1), IGF binding protein 3 (IGFBP3), and breast cancer risk: Pooled individual data analysis of 17 prospective studies. Lancet Oncol..

[25] Muti P., Quattrin T., Grant B.J., Krogh V., Micheli A., Schünemann H.J., Ram M., Freudenheim J.L., Sieri S., Trevisan M. (2002). Fasting glucose is a risk factor for breast cancer: A prospective study. Cancer Epidemiol Biomark Prev..

[26] Thomson C.A., Giuliano A., Rock C.L., Ritenbaugh C.K., Flatt S.W., Faerber S., Newman V., Caan B., Graver E., Hartz V. (2003). Measuring dietary change in a diet intervention trial: Comparing food frequency questionnaire and dietary recalls. Am. J. Epidemiol..

[27] Schroder H., Fito M., Estruch R., Martínez-González M.A., Corella D., Salas-SalvadóLamuela-Raventós J., Ros E., Salaverría I., Fiol M. (2011). A short screener is valid for assessing Mediterranean diet adherence among older Spanish men and women. J. Nutr..

[28] Rezac S., Kok C.R., Heermann M., Hutkins R. (2018). Fermented foods as a dietary source of live organisms. Front. Microbiol..

[29] Norat T., Dossus L., Rinaldi S., Overvad K., Grønbæk H., Tjønneland A., Olsen A., Clavel-Chapelon F., Boutron-Ruault M.C., Boeing H. (2007). Diet, serum insulin-like growth factor-I and IGF-binding protein-3 in European women. Eur. J. Clin. Nutr..

[30] Millward D.J. (2003). An adaptive metabolic demand model for protein and amino acid requirements. Br. J. Nutr..

[31] Young V.R., Borgonha S. (2000). Nitrogen and amino acid requirements: The Massachusetts Institute of Technology amino acid requirement pattern. J. Nutr..

[32] Musa-Veloso K., Poon T., Harkness L.S., O’Shea M., Chu Y. (2018). The effects of whole-grain compared with refined wheat, rice, and rye on the postprandial blood glucose response: A systematic review and meta-analysis of randomized controlled trials. Am. J. Clin. Nutr..

[33] Bland J.M., Altman D.G. (2011). Comparisons against baseline within randomised groups are often used and can be highly misleading. Trials.

