# Supplementary material for: A Mediterranean Dietary Intervention in Female Carriers of BRCA Mutations: Results from an Italian Prospective Randomized Controlled Trial
Source: Cancers (Basel). 2020 Dec 11;12(12):3732. doi: 10.3390/cancers12123732 (PMC7764681; doi:10.3390/cancers12123732)
Supplement: Supplementary file 1 [file cancers-12-03732-s001.pdf]

Supplementary Materials: A Mediterranean Dietary Intervention in Female Carriers of BRCA Mutations: Results from an Italian Prospective Randomized Controlled Trial

Eleonora Bruno, Andreina Oliverio, Angelo Virgilio Paradiso, Antonella Daniele, Stefania Tommasi, Antonio Tufaro, Daniela Andreina Terribile, Stefano Magno, Alessio Filippone, Elisabetta Venturelli, Daniele Morelli, Ivan Baldassari, Maria Luisa Cravana, Siranoush Manoukian and Patrizia Pasanisi

AN EXAMPLE OF DAILY MENU

BREAKFAST 1	BREAKFAST 2
Barley drink<br>Whole grain bread<br>Hazelnut cream	Bancha tea<br>Chickpea fritters<br>Jam without sugar

LUNCH
Brown rice with vegetable stew
Mackerel patè
Marinated cabbage with humeboshi vinegar
Lemon cake

SNACKS
100 g fresh fruits
20 g dried fruit

DINNER
Miso soup
Pumpkin and buckwheat dumpling
Cannellini bean salad
Carrots with ginger

	MENU with BREAKFAST 1		MENU with BREAKFAST 2		*LARN
Kcal	1705		1630		
Protein	49.6g	11%	50.9g	12%	10-15%
Total fat	58.1g	30%	54.9g	30%	25-30%
Saturated	7.8g	4%	7.3g	4%	10 %
Monounsaturated	25.8g	13%	27.2g	15%	
Polyunsaturated	14.9g	9%	14.8g	8%	5-10%
Carbohydrate	261g	58%	248g	57%	45-60%
of which sugars	47.5g	10%	46.8g	10%	< 15%
Fiber	35.1 g		35.1 g		30 g
Cholesterol	14.1 mg		14.1 mg		< 300 mg

*LARN - Levels of reference intake of nutrients and energy for the Italian population

Figure S1. An example of the proposed menu. The figure reports an example of the proposed menu (with two different breakfast) and its nutritional content. The miso soup and humeboshi vinegar are fermented foods of Japanese tradition.

Table S1. Baseline daily frequency of consumption of selected food or food groups by randomization group.

Food/Food Groups	IG (*n* = 254)	CG (*n* = 248)
Whole-grain products	1.17 ± 1.14	1.16 ± 1.33
Legumes & soy products	0.42 ± 0.61	0.34 ± 0.60
Vegetables	2.81 ± 2.07	2.68 ± 2.08
Fruits	1.52 ± 1.29	1.40 ± 1.23
Nuts & seeds	0.52 ± 0.64	0.50 ± 0.70
Refined-grain products	1.01 ± 1.10	1.06 ± 1.03
Sugary food & beverages	1.66 ± 1.45	1.92 ± 1.51
Alcoholic drinks	0.27 ± 0.51	0.32 ± 0.55
Dairy	0.93 ± 0.92	0.99 ± 0.91
Red & processed meats	0.43 ± 0.73	0.51 ± 0.66
Eggs & white meats	0.39 ± 0.67	0.39 ± 0.58
Fish	0.44 ± 0.68	0.41 ± 0.67

The table reports data from the 24-h food frequency diaries on baseline food intake.

Table S2. Analysis of differences (intention to treat analysis) in food consumption between the two groups (416 women).

MEDAS Items	IG (216)	CG (200)	p*
How much olive oil do you consume in a day (including oil used for frying or salads)?	+0.12	+0.42	0.19
How many vegetable servings do you eat per day? (1 serving ~200 g)	+0.11	+0.07	0.55
How many fruit units do you eat per day? (1 serving ~ 100–150 g)	−0.19	+0.11	0.03
How many servings of red meat, hamburger, or meat products do you eat per day?	−0.36	−0.17	0.01
How many servings of butter, margarine, cream do you eat per day? (1 serving = 12 g)	−0.11	−0.08	0.50
How many sweet beverages do you drink per day?	−0.18	−0.09	0.38
How much wine do you drink per week? (glass)	−0.11	−0.12	0.97
How many servings of legumes do you eat per week? (1 portion= 150 g)	+0.52	+0.12	0.01
How many servings of fish or shellfish do you eat per week? (1 portion= 150/200 g)	+0.28	+0.13	0.25
How many times per week do you eat commercial sweets, or pastries (not homemade), such as cakes, cookies, biscuits or custard?	−1.93	−0.64	<0.001
How many servings of nuts do you eat per week? (1 portion= 30 g)	+0.93	+0.21	0.03
How many times per week do you eat pasta?	−0.64	−0.38	0.10
MEDAS SCORE	+1.10	+0.42	<0.001

*p of ANOVA controlling for center, age, BMI at baseline, education and menstrual status
The table reports the "delta" analysis of differences in the consumption of Mediterranean food (MEDAS data).

Publisher's Note: MDPI stays neutral with regard to jurisdictional claims in published maps and institutional affiliations.

© 2020 by the authors. Licensee MDPI, Basel, Switzerland. This article is an open access article distributed under the terms and conditions of the Creative Commons Attribution (CC BY) license (http://creativecommons.org/licenses/by/4.0/).
